# Supplementary material for: SGC-CAMKK2-1: A Chemical Probe for CAMKK2
Source: Cells. 2023 Jan 11;12(2):287. doi: 10.3390/cells12020287 (PMC9856672; doi:10.3390/cells12020287)
Supplement: Supplementary file 1 [file cells-12-00287-s001.zip › File S1. Experimental details for synthesis and characterization of SGC-CAMKK2-1 and SGC-CAMKK2-1N.pdf]

# SGC-CAMKK2-1: A chemical probe for CAMKK2

Carrow Wells[1], Yi Liang[1], Thomas L. Pulliam[2], Chenchu Lin[2], Dominik Awad[2], Benjamin Eduful[1], Sean O'Byrne[1], Mohammad Anwar Hossain[1], Carolina Moura Costa Catta-Preta[3], Priscila Zonzini Ramos[3], Opher Gileadi[3], Carina Gileadi[3], Rafael M. Couñago[3], Brittany Stork[4], Christopher G Langendorf[5], Kevin Nay[5,6], Jonathan Oakhill[5], Debarati Mukherjee[7], Luigi Racioppi[8,9], Anthony Means[4], Brian York[4], Donald P. McDonnell[7], John W. Scott[5,6,10], Daniel E. Frigo[2,11,12,13] and David H. Drewry[1,14]*

[1]Structural Genomics Consortium, UNC Eshelman School of Pharmacy, University of North Carolina at Chapel Hill, Chapel Hill, NC 27599, USA.

[2]Department of Cancer Systems Imaging, The University of Texas MD Anderson Cancer Center, Houston, TX 77054, USA.

[3]Centro de Química Medicinal (CQMED), Centro de Biologia Molecular e Engenharia Genética (CBMEG), Universidade Estadual de Campinas (UNICAMP), Campinas, Brazil, 13083-886

[4]Department of Molecular and Cellular Biology, Baylor College of Medicine, Houston, TX 77030

[5]St Vincent's Institute of Medical Research, Fitzroy, Melbourne, Victoria 3065, Australia.

[6]Drug Discovery Biology, Monash Institute of Pharmaceutical Sciences, Parkville, Melbourne, Victoria 3052, Australia

[7]Department of Pharmacology and Cancer Biology, Duke University School of Medicine, Durham, North Carolina 27705

[8]Department of Medicine, Division of Hematological Malignancies and Cellular Therapy, Duke University School of Medicine, Durham, NC, 27710, USA

[9] Department of Molecular Medicine and Medical Biotechnology, University of Naples Federico II, Naples, 80131, Italy

[10]The Florey Institute of Neuroscience and Mental Health, Parkville, Melbourne, Victoria 3052, Australia

[11]Department of Genitourinary Medical Oncology, The University of Texas MD Anderson Cancer Center, Houston, TX 77030, USA.

[12]Center for Nuclear Receptors and Cell Signaling, University of Houston, Houston, TX, 77204, USA.

[13]Department of Biology and Biochemistry, University of Houston, Houston, TX 77204, USA

[14]Lineberger Comprehensive Cancer Center, Department of Medicine, School of Medicine, University of North Carolina at Chapel Hill, Chapel Hill, NC, 27599, USA.

*Correspondence: David.Drewry@unc.edu;

## General Chemistry Information

All reagents and solvents, unless specifically stated, were used as obtained from their commercial sources without further purification. Air and moisture sensitive reactions were performed under an inert atmosphere using nitrogen in a previously oven-dried or flame-dried reaction flask, and addition of reagents were done using a syringe. All microwave (MW) reactions were carried out in a Biotage Initiator EXP US 400W microwave synthesizer. Thin layer chromatography (TLC) analyses were performed using 200 μm pre-coated sorbtech fluorescent TLC plates and spots were visualized using UV light. High resolution mass spectrometry samples were analyzed with a ThermoFisher Q Exactive HF-X (ThermoFisher, Bremen, Germany) mass spectrometer coupled with a Waters Acquity H-class liquid chromatograph system. Column chromatography was undertaken with a Biotage Isolera One instrument. Nuclear magnetic resonance (NMR)

spectrometry was run on a varian Inova 400 MHz or Bruker Avance III 700 MHz spectrometer equipped with a TCI H-C/N-D 5 mm cryoprobe and data was processed using the MestReNova processor. Chemical shifts are reported in ppm with residual solvent peaks referenced as internal standard.

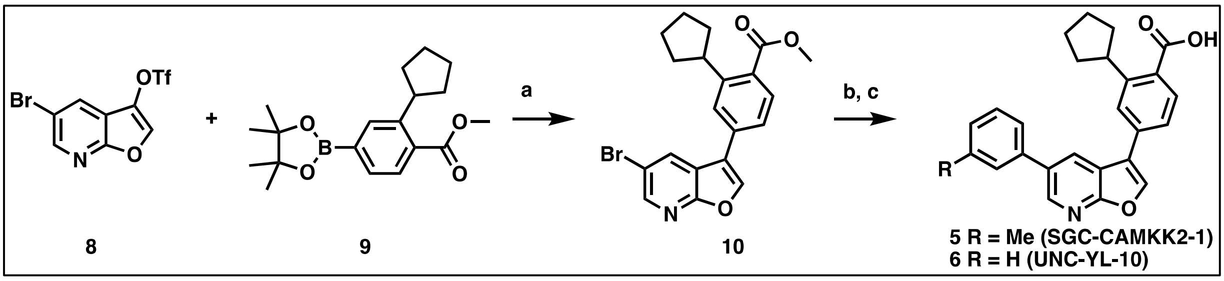


Scheme S1. Reagent and conditions: a) tetrakis (0.10 eq.), $Na_2CO_3$ (3 eq.), MeOH (2.0 mL), DCM (0.5 mL), 115 °C, 10 min, MW, 59%; b) $Pd_2(dba)_3$ (0.05 eq.), XPhos (0.01 eq.) and $Cs_2CO_3$ (3 eq.), dioxane:water (10:1, 2 mL), 120 °C, 16 h, 84%; c) 1 N NaOH, 75 °C, 4 h.

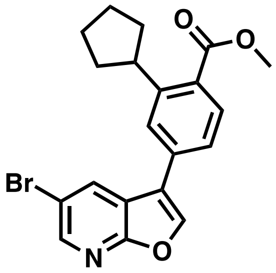


Synthesis of **methyl 4-(5-bromofuro[2,3-*b*]pyridin-3-yl)-2-cyclopentylbenzoate (10)**:

A microwave vial was charged with 5-bromofuro[2,3-b]pyridin-3-yl trifluoromethanesulfonate (250 mg, 0.72 mmol, 1 eq.) , methyl 2-cyclopentyl-4-(4,4,5,5-tetramethyl-1,3,2-dioxaborolan-2-yl)benzoate (358 mg, 1.08 mmol, 1 eq.), palladiumtetrakis (83.50 mg, 0.72 mmol, 0.10 eq.), sodium carbonate (230 mg, 2.17 mmol, 3 eq.), MeOH (2.0 mL) and DCM (0.5 mL) . The vial was sealed and irradiated in a microwave synthesizer at 115 °C for 10 min. Vial content was neutralized with aq. HCl and and extracted with ethyl acetate. The organic phases were combined and dried over sodium sulfate, concentrated and purified using flash column chromatography eluting with

EtOAc/Hexanes to afford methyl 4-(5-bromofuro[2,3-b]pyridin-3-yl)-2-cyclopentylbenzoate**, 10 (59%)** was obtained as an off-white solid. $^{1}$H NMR (400 MHz, DMSO-d6) δ 8.74 (s, 1H), 8.56 (d, J = 1.8 Hz, 1H), 8.48 (d, J = 1.6 Hz, 1H), 7.75 (d, J = 8.2 Hz, 2H), 7.69 (dd, J = 7.9, 1.7 Hz, 1H), 3.85 (s, 3H), 3.71 – 3.58 (m, 1H), 2.08 – 1.96 (m, 2H), 1.87 – 1.76 (m, 2H), 1.73 – 1.61 (m, 4H). $^{13}$C NMR (101 MHz, DMSO-$d_6$) δ 167.87, 160.49, 147.04, 144.88, 144.75, 133.42, 132.34, 130.17, 130.01, 124.98, 124.15, 119.61, 119.46, 115.29, 52.16, 41.43, 34.20, 25.19.

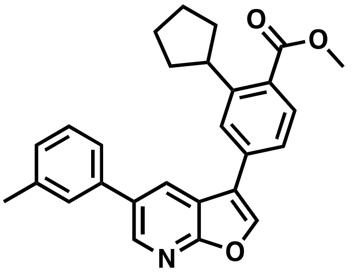

**methyl 2-cyclopentyl-4-(5-(*m*-tolyl)furo[2,3-*b*]pyridin-3-yl)benzoate:** 4-(5-bromofuro[2,3-b]pyridin-3-yl)-2-cyclopentylbenzoate (100 mg, 0.25 mmol) and 3-methylphenylboronic acid (51 mg, 0.38 mmol) were were dissolved in a 10:1 mixture of dioxane and water (2.0 mL). $Pd_2(dba)_3$ (11 mg, 0.013 mmol), XPhos (12 mg, 0.025 mmol) and cesium carbonate (244 mg, 0.75 mmol) were added. The flask was flushed with nitrogen gas and stirred at 120 ºC for 16 h. Once cooled the volatiles were removed *in vacuo*, and the crude purified by column chromatography (5% EtOAc/hexane) to afford the biaryl furopyridine (86 mg, 84%) as a white solid. 1H NMR (400 MHz, $CDCl_3$) δ 8.60 (d, J = 2.2 Hz, 1H), 8.28 (d, J = 2.2 Hz, 1H), 7.98 (s, 1H), 7.88 (dd, J = 8.1, 0.4 Hz, 1H), 7.68 (d, J = 1.8 Hz, 1H), 7.50 (dd, J = 8.0, 1.8 Hz, 1H), 7.45 – 7.37 (m, 3H), 7.24 (dtd, J = 6.6, 1.8, 0.7 Hz, 1H), 3.93 (s, 3H), 3.91 – 3.81 (m, 1H), 2.46 (d, J = 0.6 Hz, 3H), 2.23 – 2.12 (m, 2H), 1.93 – 1.82 (m, 2H), 1.80 – 1.72 (m, 2H), 1.70 – 1.62 (m, 2H).

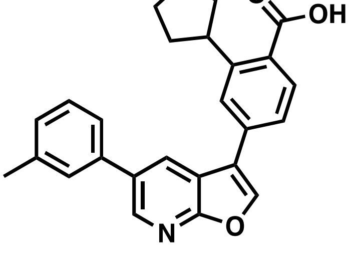

**2-cyclopentyl-4-(5-(*m*-tolyl)furo[2,3-*b*]pyridin-3-yl)benzoic acid (5):** A methyl 2-cyclopentyl-4-(5-(m-tolyl)furo[2,3-b]pyridin-3-yl)benzoate (50 mg) in methanol was treated with 1 N NaOH (aq.) and stirred for 4 hours at 75 °C. Upon completion of the reaction the reaction mixture was acidified with 2 N HCl (aq.). Pure product was extracted with ethyl acetate and crystalized from DCM/hexane. 1H NMR (400 MHz, DMSO-$d_6$) δ 12.93 (s, 1H), 8.69 (s, 1H), 8.63 (d, J = 2.2 Hz, 1H), 8.49 (d, J = 2.2 Hz, 1H), 7.85 – 7.71 (m, 3H), 7.65 – 7.49 (m, 2H), 7.39 (t, J = 7.6 Hz, 1H), 7.29 – 7.19 (m, 1H), 3.89 – 3.73 (m, 1H), 2.39 (s, 3H), 2.14 – 1.95 (m, 2H), 1.81 (d, J = 11.5 Hz, 2H), 1.75 – 1.56 (m, 4H). $^{13}$C NMR (176 MHz, DMSO-$d_6$) δ 169.73, 162.05, 147.59, 144.26, 143.69, 138.83, 137.89, 134.13, 133.46, 131.44, 130.77, 129.49, 128.94, 128.59, 125.54, 125.00, 124.60, 120.67, 118.08, 41.65, 34.86, 25.81, 21.55. HRMS m/z: $[M+H]^+$ Calc'd for $C_{26}H_{23}NO_3$ 398.17; Found 398.16.

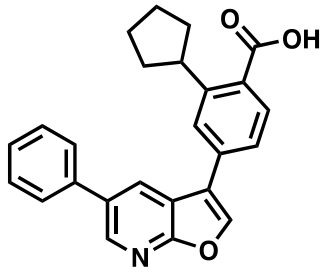


Synthesis of **2-cyclopentyl-4-(5-phenylfuro[2,3-b]pyridin-3-yl)benzoic acid**, **(6)**: A mixture of 4-(5-bromofuro[2,3-b]pyridin-3-yl)-2-cyclopentylbenzoic acid (48.0 mg, 0.12 mmol), phenylboronic acid (30.0 mg, 0.25 mmol), sodium carbonate (40.0 mg, 0.37 mmol) and $Pd(PPh_3)_4$ (14.0 mg, 12 µmol) in a mixture of Dioxane (2 mL) and Water (0.5 mL) was heated to 90 °C under nitrogen overnight. Contents were neutralized with aq. HCl and extracted with ethyl acetate. The organic phases were combined, dried over anhydrous sodium sulfate, concentrated and purified using flash column chromatography eluting with EtOAc/Hexanes to obtain the target compound, 2-cyclopentyl-4-(5-

phenylfuro[2,3-b]pyridin-3-yl)benzoic acid **6** (39 mg, 82 %) as a white solid. $^{1}$H NMR (400 MHz, DMSO-d6) δ 12.95 (s, 1H), 8.72 (s, 1H), 8.67 (d, J = 2.2 Hz, 1H), 8.53 (d, J = 2.2 Hz, 1H), 7.82 (d, J = 1.5 Hz, 1H), 7.81 – 7.79 (m, 4H), 7.56 – 7.50 (m, 2H), 7.47 – 7.41 (m, 1H), 3.86 – 3.77 (m, 1H), 2.11 – 2.01 (m, 2H), 1.87 – 1.79 (m, 2H), 1.73 – 1.62 (m, 4H). $^{13}$C NMR (101 MHz, DMSO-d6) δ 169.27, 161.59, 147.04, 143.81, 143.24, 137.50, 133.62, 132.88, 131.00, 130.27, 129.10, 128.18, 127.83, 127.46, 125.02, 124.13, 120.20, 117.61, 41.22, 34.35, 25.28. HRMS m/z: $[M+H]^+$ Calc'd for $C_{25}H_{21}NO_3$ 384.1600; Found 384.1593. MP Range 259-262.

Intermediate **9** was synthesized as previously described by Eduful et al.[1] Briefly **11** was treated with cylclopentylmagnesium bromide to afford intermediate **12**. The pinocol borane was then installed under standard conditions to obtain **9**.

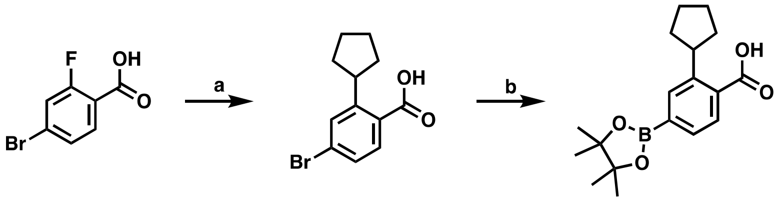


11 12 9

Scheme S2. Synthetic route to obtain key intermediate **9**: Reagents and conditions: a) Cyclopropylmagnesium bromide, THF, -10 to 25 °C, 5 h, then aq. HCl; b) $Pd(dppf)Cl_2$.DCM (0.05 eq.), KOAc, bis(pinacolato)diboron (2 eq.), 1,4-dioxane, 100 °C, 2 h

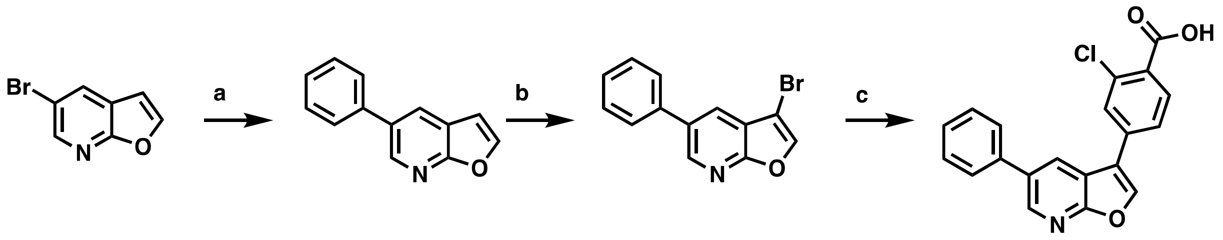


13 14 15 7

Scheme S3. Synthesis of compound 7. Reagents and conditions: a) tetrakis (0.10 eq.), $Na_2CO_3$ (3 eq.), DMF (20.0 mL), water (5.0 mL), 90 °C, 16 h, heating, 68%; b) DCM, $Br_2$, 0 °C to r.t, 1 h , 44%; c) tetrakis (0.10 eq.), $Na_2CO_3$ (3 eq.), dioxane (10.0 mL), water (2.5 mL), 90 °C, 16 h, heating, 83%

**5-phenylfuro[2,3-*b*]pyridine (14):**

A mixture of 5-bromofuro[2,3-*b*]pyridine (1.00 g, 5.05 mmol), phenylboronic acid (924 mg, 7.57 mmol), palladiumtetrakis (292 mg, 0.25 mmol), and cesium carbonate (3.29 g, 10.1 mmol) in DMF (20 mL) and Water (5.0 mL) under nitrogen atmosphere was heated to 90 $^{o}$C for 16 h. The reaction mixture was allowed to cool to room temperature and water (100 mL) was added and extracted into EtOAc (150 mL, 2X). The combined organic layer was washed with brine (20 mL), dried over sodium sulfate, and concentrated in vacuo. The residue was purified by silica gel column chromatography (0-20% EtOAc/hexanes), to afford 5-phenylfuro[2,3-*b*]pyridine, **14** (0.67 g, 68 %) as a pale solid. LCMS $[M+H]^+$: *m/z* 195.08.

**3-bromo-5-phenylfuro[2,3-b]pyridine (15):**

To a mixture of 5-phenylfuro[2,3-*b*]pyridine (630 mg, 3.23 mmol) in $CHCl_3$ (25 mL) at 0 °C was slowly added bromine (183 μL, 3.55 mmol) and the mixture was stirred at room temperature for 1.5 hr. The organic layer was washed with 1 M sodium sulfite and brine, dried over sodium sulfate, and concentrated in vacuo to give 2,3-dibromo-5-phenylfuro[2,3-*b*]pyridine. To a solution of 2,3-dibromo-5-phenylfuro[2,3-*b*]pyridine in THF (20 mL) at 0 °C was added a mixture of NaOH (182 mg, 85% Wt, 3.87 mmol) in MeOH (5 mL), and the mixture was stirred for 30 min. The reaction mixture was diluted

with EtOAc, and the organic layer was washed with water and saturated aqueous sodium hydrogen carbonate, dried over sodium sulfate, and concentrated in vacuo. The residue was purified by silica gel column chromatography eluting with 0 - 15% EtOAc/hexanes to afford 3-bromo-5-phenylfuro[2,3-*b*]pyridine, **15** (390 mg, 44%) as a brownish solid. LCMS $[M+H]^+$: *m/z* 272.12/274.12.

**2-chloro-4-(5-phenylfuro[2,3-b]pyridin-3-yl)benzoic acid (7):**

A mixture of 3-bromo-5-phenylfuro[2,3-*b*]pyridine (350 mg, 1.28 mmol), 4-borono-2-chlorobenzoic acid (435 mg, 2.17 mmol), palladiumtetrakis (103 mg, 89.4 μmol) and sodium carbonate (541 mg, 5.11 mmol) in dioxane (10 mL) and water (2.5 mL) under nitrogen atmosphere was heated to 90 °C for 16 h. To the reaction mixture was added water (50 mL) and extracted into EtOAc (80 mL, 2x). The combined organic layer was washed with brine (20 mL), dried over sodium sulfate, and concentrated in vacuo. The residue was purified by silica gel column chromatography (45% EtOAc/hexanes), to afford 2-chloro-4-(5-phenylfuro[2,3-*b*]pyridin-3-yl)benzoic acid **7** (0.37 g, 1.1 mmol, 83 %) as a near yellow solid. $^1$H NMR (400 MHz, DMSO-$d_6$) δ 13.43 (s, 1H), 8.79 (s, 1H), 8.66 (dd, *J* = 17.0, 2.2 Hz, 2H), 8.04 – 7.98 (m, 2H), 7.94 (d, *J* = 8.1 Hz, 1H), 7.86 – 7.82 (m, 2H), 7.56 – 7.51 (m, 2H), 7.47 – 7.42 (m, 1H). $^{13}$C NMR (176 MHz, DMSO-$d_6$) δ 166.80, 162.00, 145.21, 143.95, 137.87, 135.65, 133.59, 133.18, 132.34, 130.47, 129.54, 128.95, 128.94, 128.34, 128.11, 126.04, 119.28, 117.61. HRMS calcd for $C_{20}H_{12}ClNO_3$ $[M+H]^+$: *m/z* 350.0584; found 350.0568.

## $^{1}$H NMR for **5**

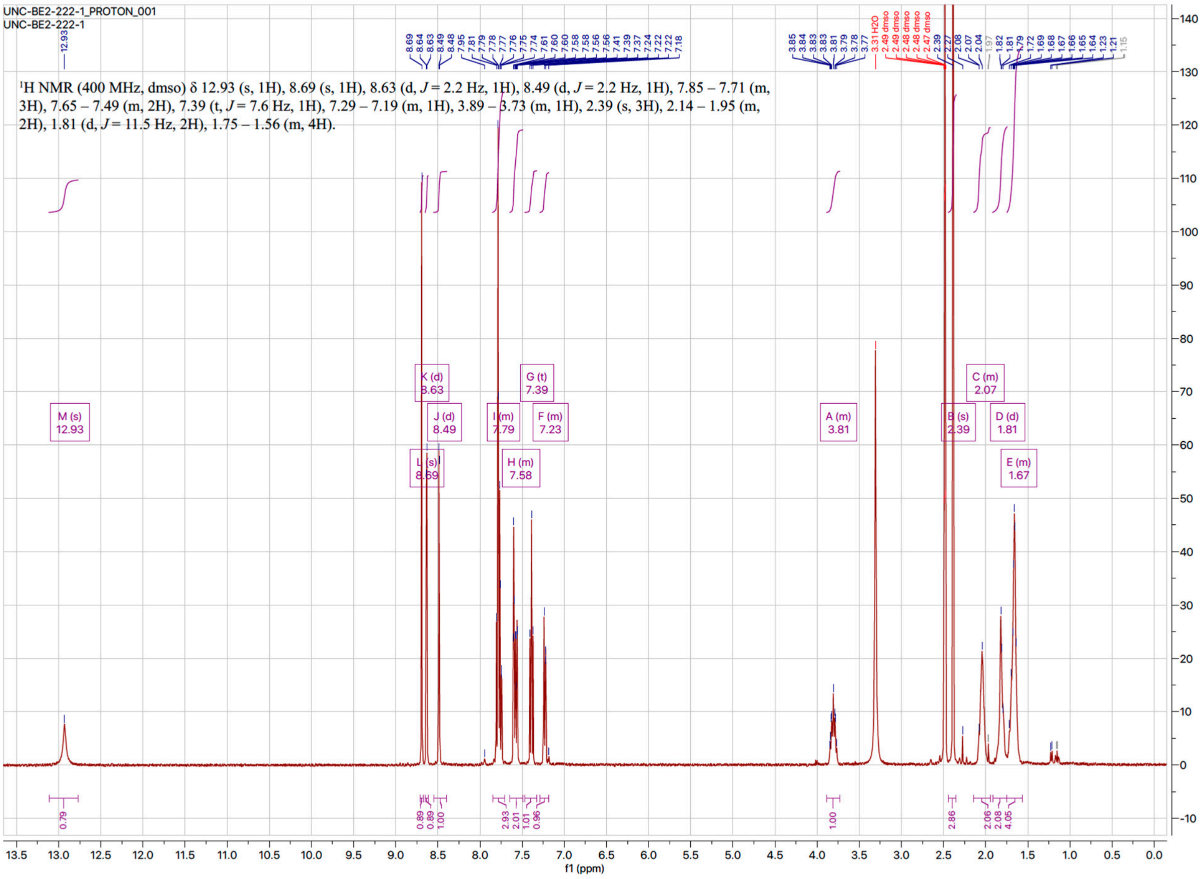

UNC-BE2-222-1_PROTON_001
UNC-BE2-222-1
¹H NMR (400 MHz, dmso) δ 12.93 (s, 1H), 8.69 (s, 1H), 8.63 (d, J = 2.2 Hz, 1H), 8.49 (d, J = 2.2 Hz, 1H), 7.85 – 7.71 (m, 3H), 7.65 – 7.49 (m, 2H), 7.39 (t, J = 7.6 Hz, 1H), 7.29 – 7.19 (m, 1H), 3.89 – 3.73 (m, 1H), 2.39 (s, 3H), 2.14 – 1.95 (m, 2H), 1.81 (d, J = 11.5 Hz, 2H), 1.75 – 1.56 (m, 4H).
M (s) 12.93
L (s) 8.69
K (d) 8.63
J (d) 8.49
I (m) 7.79
H (m) 7.58
G (t) 7.39
F (m) 7.23
A (m) 3.81
B (s) 2.39
C (m) 2.07
D (d) 1.81
E (m) 1.67
3.31 H2O
2.49 dmso
f1 (ppm)

$^{13}$C NMR for **5**

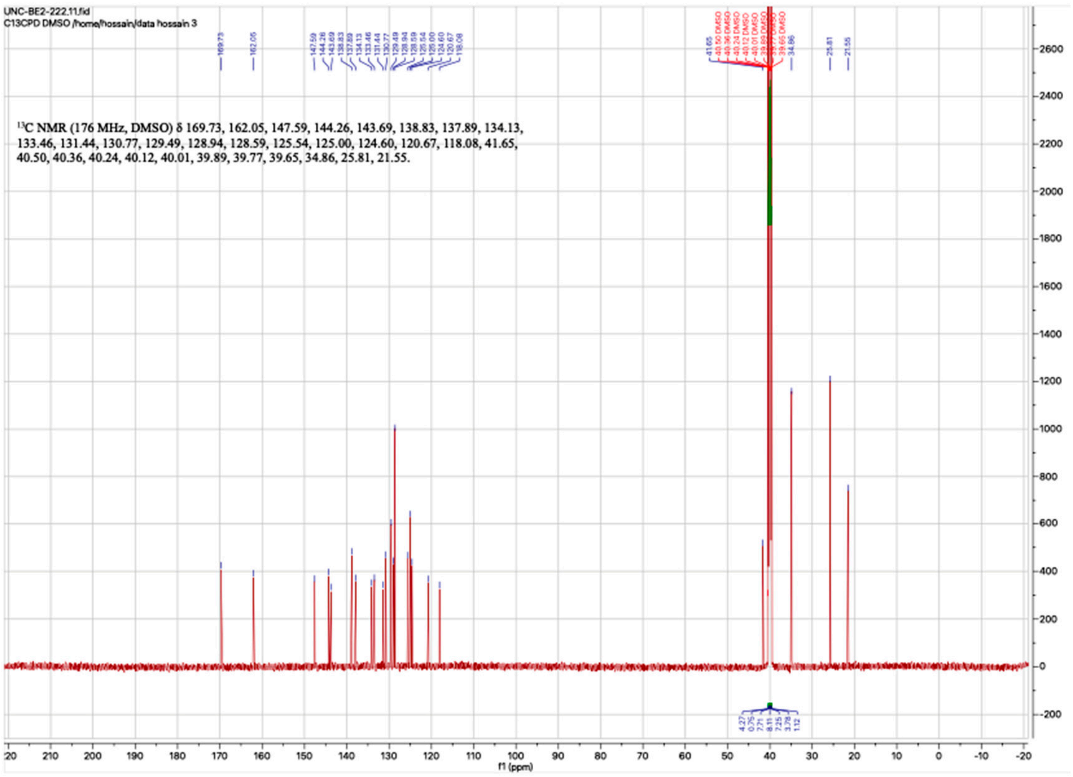

UNC-BE2-222.11.fid
C13CPD DMSO /home/hossain/data hossain 3
$^{13}$C NMR (176 MHz, DMSO) δ 169.73, 162.05, 147.59, 144.26, 143.69, 138.83, 137.89, 134.13, 133.46, 131.44, 130.77, 129.49, 128.94, 128.59, 125.54, 125.00, 124.60, 120.67, 118.08, 41.65, 40.50, 40.36, 40.24, 40.12, 40.01, 39.89, 39.77, 39.65, 34.86, 25.81, 21.55.
f1 (ppm)

## $^1$H NMR for **6**

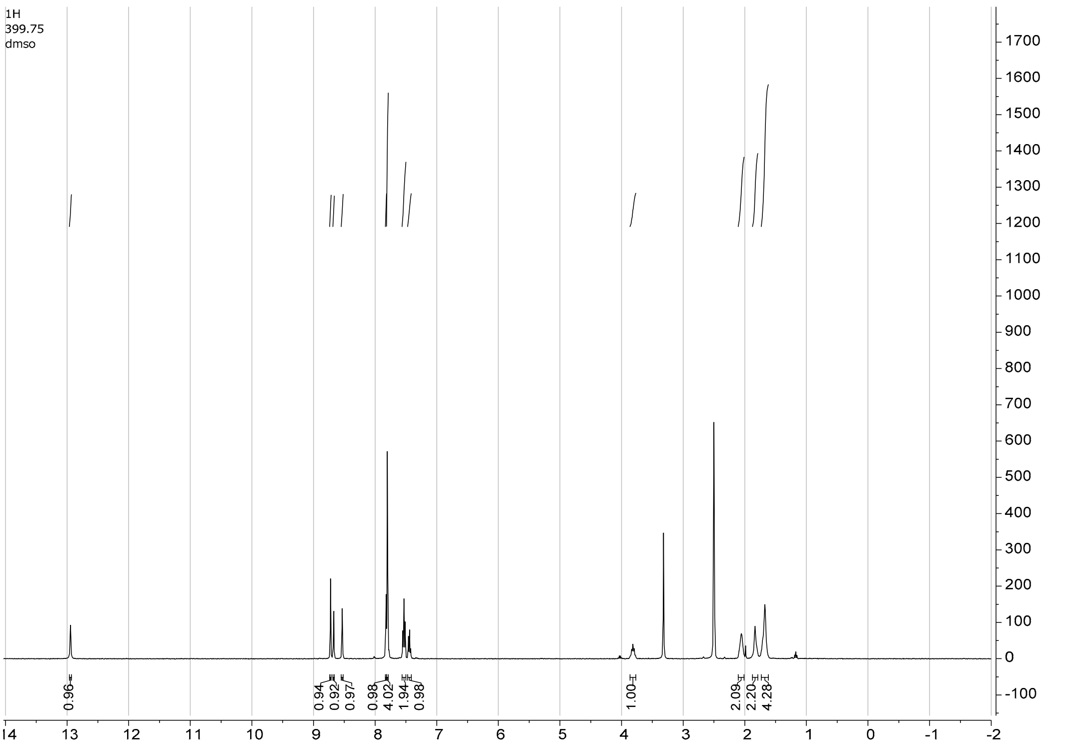

$^{13}C$ NMR for **6**

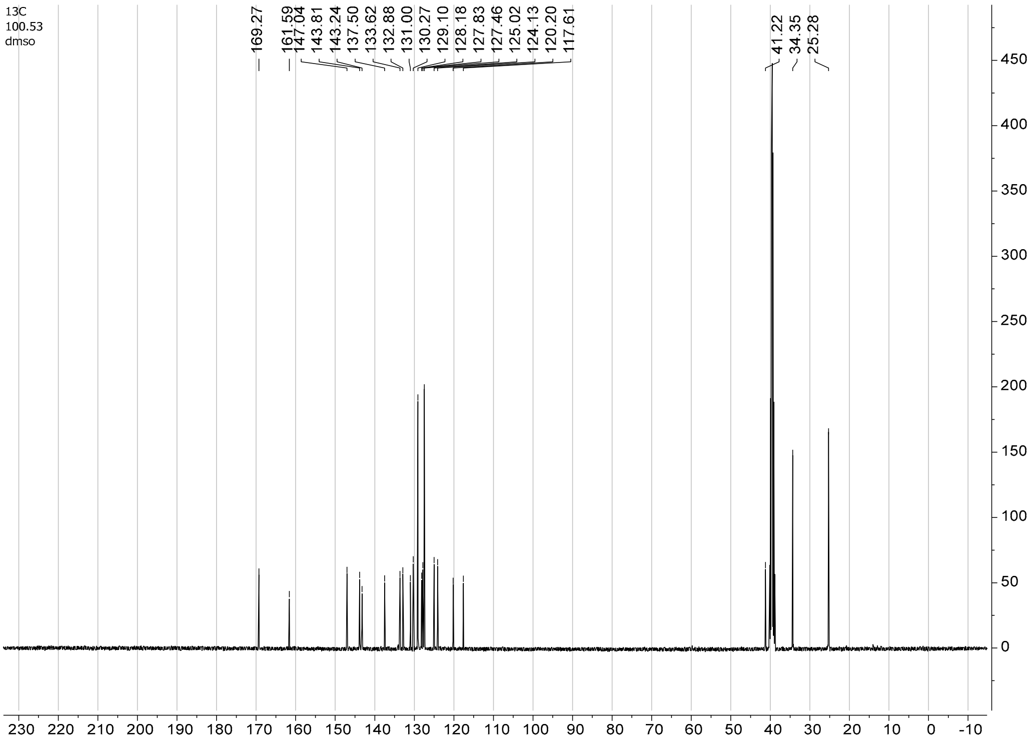

13C
100.53
dmso
169.27
161.59
147.04
143.81
143.24
137.50
133.62
132.88
131.00
130.27
129.10
128.18
127.83
127.46
125.02
124.13
120.20
117.61
41.22
34.35
25.28
450
400
350
300
250
200
150
100
50
0
230 220 210 200 190 180 170 160 150 140 130 120 110 100 90 80 70 60 50 40 30 20 10 0 -10

$^{1}H$ NMR for **7**

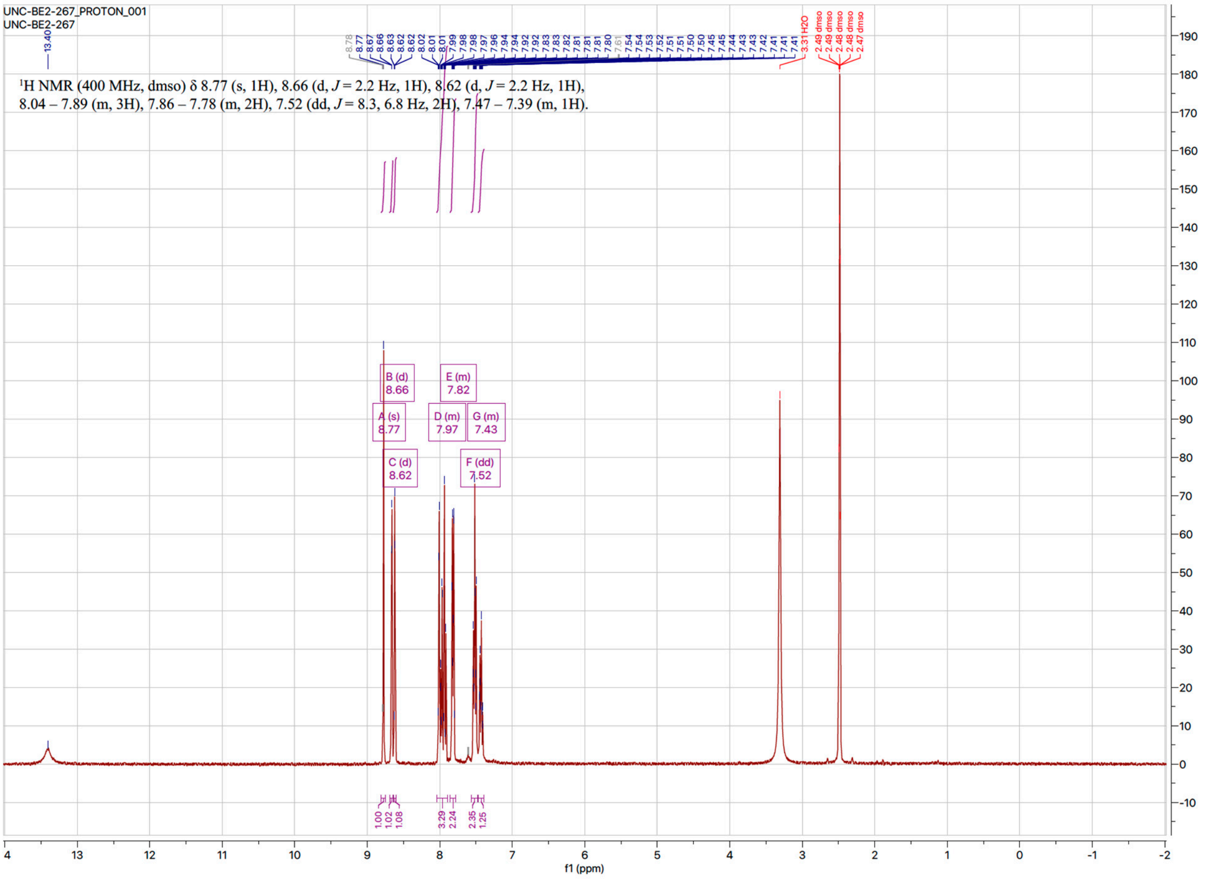

UNC-BE2-267_PROTON_001
UNC-BE2-267
¹H NMR (400 MHz, dmso) δ 8.77 (s, 1H), 8.66 (d, J = 2.2 Hz, 1H), 8.62 (d, J = 2.2 Hz, 1H), 8.04 – 7.89 (m, 3H), 7.86 – 7.78 (m, 2H), 7.52 (dd, J = 8.3, 6.8 Hz, 2H), 7.47 – 7.39 (m, 1H).
f1 (ppm)

$^{13}$C NMR for **7**

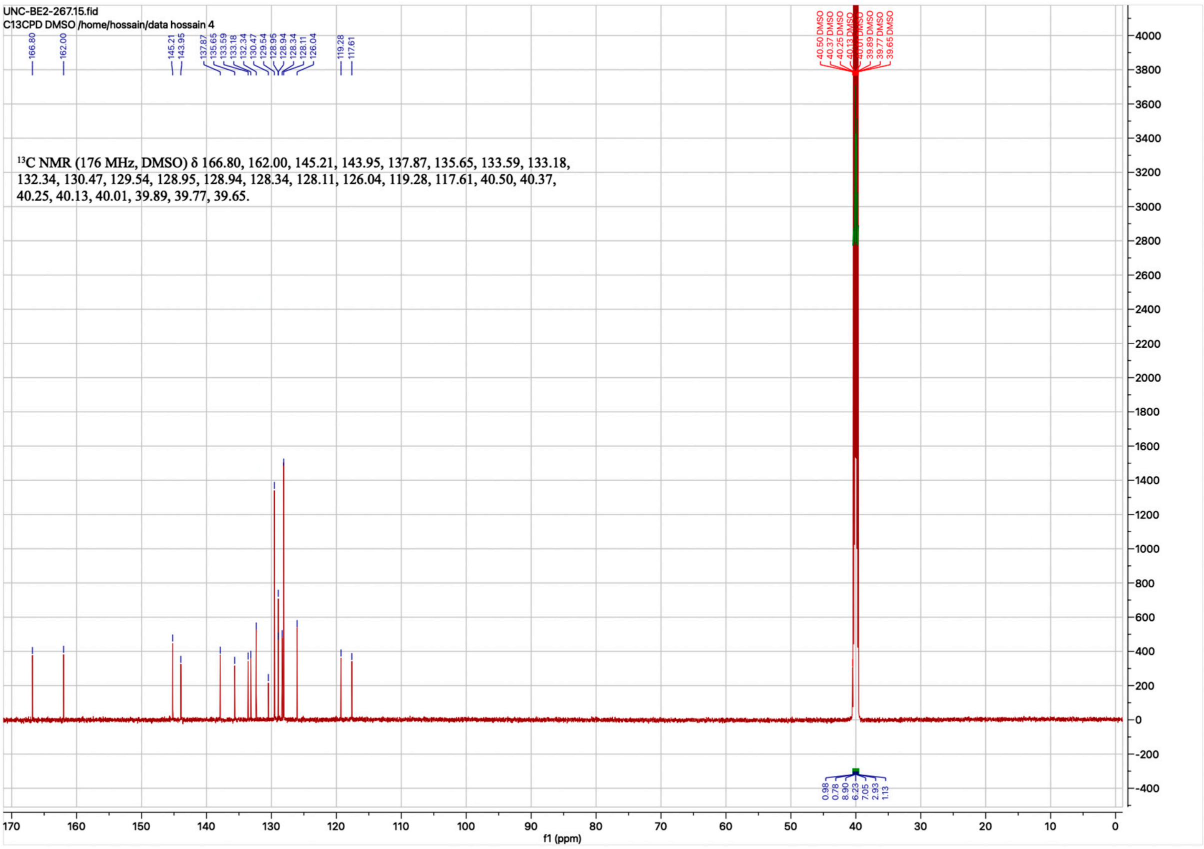


1. Eduful, B.J.; O'Byrne, S.N.; Temme, L.; Asquith, C.R.M.; Liang, Y.; Picado, A.; Pilotte, J.R.; Hossain, M.A.; Wells, C.I.; Zuercher, W.J.; Catta-Preta, C.M.C.; Zonzini Ramos, P.; Santiago, A.S.; Counago, R.M.; Langendorf, C.G.; Nay, K.; Oakhill, J.S.; Pulliam, T.L.; Lin, C.; Awad, D.; Willson, T.M.; Frigo, D.E.; Scott, J.W.; Drewry, D.H. Hinge Binder Scaffold Hopping Identifies Potent Calcium/Calmodulin-Dependent Protein Kinase Kinase 2 (CAMKK2) Inhibitor Chemotypes. *Journal of medicinal chemistry* **2021**, *64*, 10849-10877, doi:10.1021/acs.jmedchem.0c02274.
